# Risk of Depression, Anxiety, and Stress During the Second Wave of COVID-19 in Slovenia

**DOI:** 10.3389/fpsyt.2021.788898

**Published:** 2022-01-12

**Authors:** Polona Rus Prelog, Teodora Matić, Peter Pregelj, Aleksander Sadikov

**Affiliations:** ^1^Centre for Clinical Psychiatry, University Psychiatric Clinic Ljubljana, Ljubljana, Slovenia; ^2^Artificial Intelligence Laboratory, Faculty of Computer and Information Science, University of Ljubljana, Ljubljana, Slovenia; ^3^Faculty of Medicine, University of Ljubljana, Ljubljana, Slovenia

**Keywords:** depression, anxiety, stress, COVID-19, second wave, health workers

## Abstract

The spread of Severe Acute Respiratory Syndrome Coronavirus 2 (SARS-CoV-2) causing coronavirus disease 2019 (COVID-19) pandemic has led to numerous negative consequences on the mental health of the population throughout the world. The main aim of our study was to compare the risk for depression, anxiety, and stress during the second wave of the pandemic in Slovenia. An additional goal was to analyze the association of depression, anxiety, and stress, with the most relevant subjective factors that define the quality of life. Furthermore, we aimed at determining whether health workers have a higher risk for depression following the course of the pandemic. The study was conducted on the general population, between July 2020 and January 2021 through an online survey. The data of 1,728 respondents in two samples of respondents (782 at baseline – first measurement point and 946 during the second measurement point) of the second wave were analyzed using zero-inflated negative binomial regression and Mann-Whitney *U*-test. The findings of this study show that the rise the second wave was associated with a higher risk for depression, anxiety and stress. The risk for all three was higher for younger participants. Women showed a higher risk for anxiety and stress. Finances, relationships, and housing dissatisfaction were relevant predictors for depression, anxiety and stress. Health workers in our sample showed a higher risk for stress, but not for depression or anxiety, than the general population. Our findings highlight the urgent need for coordinating and developing mental health services and tailored interventions to reduce the mental health burden, especially in the younger.

## Introduction

The spread of Severe Acute Respiratory Syndrome Coronavirus 2 (SARS-CoV-2) causing coronavirus disease 2019 (COVID-19) pandemic has led to numerous negative consequences on the economic, social, and healthcare system and has affected the physical and mental health of the population throughout the world (1). Almost all countries have adopted (intermittent) confinement measures, including lockdown, home isolation, and physical distancing. When rates of infections were high, people were bound to their homes and their living arrangement; contacts were limited to a minimum, and closest personal relationships gained importance. Many studies have already examined the effects of the pandemic, mainly in the early phase of the pandemic. The spread of infections, the direct impact of the disease and the adaptation to the associated disease containment measures were proposed to influence the mental health of the population as well (2–4). Highly significant levels of psychological distress that, in many cases, would meet the threshold for clinical relevance, especially for anxiety and depression, were found (5).

Considerably fewer studies analyzed depression during the second wave of the COVID pandemic (6–8), and we still lack data from longitudinal studies. Fukase showed that during prolonged psychological distress caused by the COVID-19 pandemic, the prevalence of depressive symptoms in Japan was two to nine times as high during the second wave as before the pandemic, even though Japan was not a lockdown country (9). A Polish study found that during the second wave, 20% of the sample had symptoms of anxiety disorders, and almost 19% had anxiety and depression symptoms (7). Depressive symptoms are often triggered by stressors, such as suboptimal living conditions. These are mainly defined by the most important personal relationships, economic and financial situations, and housing conditions. It was found that housing can be a relevant factor influencing psychological distress and the risk of depression among residents (10, 11). Economic situation and financial difficulties are also known to be associated with depression, stress, and anxiety (12, 13). During the Great Recession of 2008 in the United States, for instance, financial, housing, or employment issues were positively associated with increased anxiety and depression up to 3–4 years post-recession.

Associations between mental health and relationship quality were shown in several previous studies (14, 15). Relationship state can contribute to stress, anxiety and depression. According to prospective analyses, relationship satisfaction instability is associated with depressive symptoms and may precede, rather than follow, elevated depressive symptoms (14). During the COVID-19 pandemic, supportive close relationships were found to be among the most important predictors of health and well-being, and high-quality relationships an important resource for coping with COVID-19-related stressors (16). Studies have also shown that people who experience prolonged financial strain, lack of social connection and higher levels of stress are at risk for relationship dissatisfaction (17). During social distancing, when people are confined to their homes and to closest relationships, the subjective perceptions of all of these factors gain even more importance and influence the mental well-being.

However, not many studies have examined the mental health of the population following the rising rates of infections and restrictive measures during the subsequent waves of the pandemic, and we still lack the knowledge of psychological changes and mental health symptoms, whether they intensify or weaken as the pandemic is spreading and declining and we adapt to specific pandemic-related restrictions. Analyzing the mental health during the second and subsequent waves of the pandemic is, therefore, necessary to improve our understanding of these dynamic changes and their impact on mental health. Understanding of depression and anxiety symptoms' risk factors is relevant for mental health services planning and accurate strategies development during this and subsequent pandemics.

Given the fast and exponential spread of infections with concomitant prolonged and more restrictive measures during the second wave in Slovenia, we could expect the mental health impact may shift over the course of the pandemic.

In this context, our main aim was to evaluate and compare the risk for depression, anxiety, and stress during the second wave of the pandemic. At baseline, when the numbers of infected were low, and the corresponding government measures were mild, without strict limitations; and at the second measurement point, during the second wave, when the numbers were rising rapidly (exponentially) and the country was closing down. We specifically aimed to assess which groups (as allowed per the available data) were most at risk. Additionally, we aimed to analyze the association of depression, anxiety, and stress (DAS) with important subjective factors that define the quality of life, especially in quarantine—perceived satisfaction with finances, relationships, and housing. Furthermore, we aimed at determining whether health workers, as the most exposed group, have a higher risk for DAS during the second wave of the pandemic.

## Materials and Methods

Here we briefly describe the situation in Slovenia to enlighten the timeline of events and the corresponding numbers given below. The first infection with the novel coronavirus in Slovenia was confirmed on 4 March 2020. Measures were strict and a lockdown period lasted for 12 weeks. In the first wave of the pandemic, the daily record number of positive cases was 61. The country of 2.1 million inhabitants officially declared the end of the COVID-19 epidemic in Slovenia on 31 May 2020, and life almost returned to normal for the general population; only a relatively small number of new infections were recorded during the following summer. The first wave of the pandemic was not detrimental to the Slovenian healthcare system. Slovenia's initial handling of the coronavirus outbreak was even cited as a significant success and was among the most effective in handling the COVID-19 outbreak when Europe faced the first wave of the pandemic. However, in October 2020, the disease spread rapidly, the number of cases among the population rose exponentially, and the epidemic was again announced on 18 October 2020 with strict measures following; in the second wave, the numbers were much higher—up to 2,500 of infected people per day. Slovenia was one of the hardest-hit countries during the second wave, with the highest death rate per capita in the world in December 2020 (18).

### Study Design and Participants

This study was a part of a large international multicenter study that started in Italy during the first wave of the pandemic (19). We used the study protocol questionnaire adapted for the Slovenian population. The study in Slovenia was conducted after the lockdown phase of the pandemic in July 2020. We continued the distribution until the middle of January 2021.

An online survey was set up through Google Docs and officially launched on 23 July 2020. It lasted until the middle of January 2021 in a large community sample of the Slovenian adult population (*N* = 1,790). It was implemented through a multistep procedure: (a) email invitation to healthcare professionals through their institutions, (b) social media channels (Facebook, LinkedIn) with snowball sampling strategy focused on recruiting the general population living in Slovenia during the pandemic of COVID-19), (c) mailing lists of universities and (d) other official websites or mailing lists (e.g., healthcare or welfare authorities websites, companies, etc.). It took ~20 min to complete. The study was approved by the Republic of Slovenia National Medical Ethics Committee under protocol No. 0120-283/2020/7.

The survey was advertised through all the above channels, particularly at two specific time points: at the survey launch (at baseline) and at the peak of the second wave, when numbers of infections were extremely high. That resulted in two large peaks of respondents and, while cross-sectional in nature, we were able to compare between the two time points of the second wave, first, at baseline and second, when the number of infections had risen.

There were in total 1,790 respondents—of that 782 at baseline (first measurement point), 946 during the second measurement point of Wave 2, while 62 respondents were excluded from the analysis due to responding in the so-called buffer zone. The buffer zone was defined to divide the two measurement points of the second wave and consists of all respondents who filled the survey between 1 October and 18 October 2020 (when the number of infections was very high and the epidemic was again formally declared). Therefore, 1,728 respondents were included in the final analysis. The median age of all respondents was 38 years (37 at the first measurement point and 39 in the second), with 79.9% of respondents being female (80.4% at the first and 79.5% at the second measurement point), and 31.8% of respondents being health workers (40.5% at the first, and 24.5% at the second measurement point). A detailed overview of the respondents and their characteristics is given in Table 1. The two samples (first and second measurement point) exhibit statistical differences (except for gender); these are mostly due to large sample sizes.

**Table 1:** The overview of the respondents and their characteristics.

		**Total sample**	**Time point 1**	**Time point 2**	***P*-value**
Sample size	*N*	1,728	782	946	–
Age	Mean (St.dev.)	39.5 (±11.6)	39.0 (±12.6)	40.0 (±10.7)	<0.001
	Median	38	37	39	
	Range	19–77	19–77	19–77	
Gender	Female/male	*N* = 1.381 (79.9%)/ *N* = 347 (20.1%)	*N* = 629 (80.4%)/ *N* = 153 (19.6%)	*N* = 752 (79.5%)/ *N* = 194 (20.5%)	0.669
Health worker	Yes	*N* = 549 (31.8%)	*N* = 317 (40.5%)	*N* = 232 (24.5%)	<0.001
Satisfaction with finances	Mean (St.dev.)	4.57 (±1.27)	4.50 (±1.24)	4.63 (±1.30)	<0.001
Satisfaction with relationships	Mean (St.dev.)	5.28 (±1.06)	5.32 (±1.10)	5.24 (±1.02)	<0.001
Satisfaction with housing	Mean (St.dev.)	5.49 (±1.09)	5.47 (±1.12)	5.51 (±1.06)	<0.001

### Sociodemographic Variables and Assessment Tools

Sociodemographic variables included age, gender, and information on being a healthcare worker or not. The variables related to satisfaction since the beginning of the COVID-19 pandemic were assessed with three questions regarding the perceived satisfaction with (1) finances (e.g., “Following the pandemic, how satisfied are you with your financial situation?”), (2) relationships with people they lived with during the pandemic, or relationships with their closest people if they lived alone, and (3) housing. All three variables were assessed on a 7-point Likert scale (ranging from “could not be worse” to “could not be better”). These three variables were used individually as they do not form a predefined scale.

The emotional states of depression, anxiety, and stress were assessed using the Depression, Anxiety and Stress Scale−21 Items (DASS-21), which is a set of three self-report scales and a valid tool in assessing mental health in the general population (20). All three scales showed excellent reliability, with Cronbach alpha values of 0.90, 0.86, and 0.92 for the Depression, Anxiety and Stress scale, respectively. The scores for each subscale are divided into categories ranging from normal to extremely severe, and these categories were used for a clearer representation of the results, while the regression models used continuous values (which offer more information).

### Statistical Methods

The results were summarized using descriptive statistics. Differences in depression, anxiety, stress, age, satisfaction with finances, relationships and housing between the two measuring points of the second wave were evaluated using the Mann-Whitney *U*-test, while for the differences in age and proportion of health workers, we used the chi-square test. In order to assess the impact of different factors on depression, anxiety, and stress levels, we built separate regression models for each of the outcomes. Because of overdispersion and a large number of zeros (people who show no indication of a particular symptom), we chose zero-inflated negative binomial (ZINB) regression for this task. Although our outcomes are not counts, they are integer data with no negative values and a strong positive skew, and count regression models, like the one we used, are recommended for modeling such data (21). The relative fit of the Poisson, negative binomial, and ZINB regression models was assessed using AIC and Vuong test for non-nested models with AIC and Schwarz correction (22), with the ZINB model having the best fit. ZINB regression models are two-component mixture models combining a point mass at zero with a negative binomial distribution (23). The population is modeled as consisting of a subpopulation not at risk (in further text “structural zeros”), and a subpopulation at risk for developing DASS symptoms during the study period. The model consists of two components: a negative binomial component that accounts for the at-risk population (in further text “count model part”), and a logit model accounting for structural zeros (in further text “zero-inflation model part”) (24). ZINB regression was implemented using the *pscl* package (23), while the likelihood ratio tests were implemented using the *lmtest* package (25) in R (26).

We introduced the predictor variables using the stepwise procedure, testing the improvement in fit with the likelihood ratio test after introducing each predictor. The predictors were the same for both components of the ZINB model. As the factor of primary interest, the “Time point” variable was always kept in the model, while the other predictors were kept only if their addition led to a significant improvement in model fit (as indicated by the likelihood ratio test being significant at *p* ≤ 0.05 level). Also of note is that always including the “Time point” variable in the models adjusts for the observed differences in other variables between the two measurement points.

## Results

### DASS Scores

The distribution and comparison of DASS scores between the measurement points are given in Table 2. A more detailed distribution over categories is shown in Table 3. A 36.9% (*N* = 638) of participants scored above the threshold for depression and reported at least mild depressive symptoms; 35.2% (*N* = 275) at baseline and 38.4% (*N* = 363) at the second time point. Symptoms of depression were mild to moderate in 24.9% (*N* = 431) of respondents and severe or extremely severe in 12.0% (*N* = 207); anxiety symptoms were mild to moderate in 16.5% (*N* = 285) of respondents and severe or extremely severe in 12.6% (*N* = 218); stress symptoms were at least moderate in 23.3% (*N* = 403) of people in our sample. Table 3 contains the detailed distributions of DASS scores between the measurement points.

**Table 2:** The distribution of DASS depression, DASS anxiety, and DASS stress scores.

		**Time point 1 (*N* = 782)**	**Time point 2 (*N* = 946)**	***P*-value**
DASS depression	Mean (St.dev.)	8.19 (±9.30)	8.79 (±8.90)	<0.001
	Median	4.00	6.00	
DASS anxiety	Mean (St.dev.)	5.17 (±7.21)	6.10 (±7.57)	<0.001
	Median	2.00	4.00	
DASS stress	Mean (St.dev.)	11.02 (±10.41)	11.64 (±10.02)	<0.001
	Median	8.00	10.00	

**Table 3:** Percentages of the sample with a particular category of DASS depression, DASS anxiety, and DASS stress scores.

		**Time point (*N* = 782)**	**Time point 2 (*N* = 946)**
DASS depression	Normal (0–9)^*^ N = 1,090	64.8% *N* = 507	61.6% *N* = 583
	Mild (10–12) *N* = 178	9.7% *N* = 76	10.8% *N* = 102
	Moderate (13–20) *N* = 253	13.3% *N* = 104	15.8% *N* = 149
	Severe (21–27) *N* = 109	6.4% *N* = 50	6.2% *N* = 59
	Extremely severe (28–42) *N* = 98	5.8% *N* = 45	5.6% *N* = 53
DASS anxiety	Normal (0–6) *N* = 1,225	73.3% *N* = 573	68.9% *N* = 652
	Mild (7–9) *N* = 84	5.1% *N* = 40	4.7% *N* = 44
	Moderate (10–14) *N* = 201	10.1% *N* = 79	12.9% *N* = 122
	Severe (15–19) *N* = 91	5.1% *N* = 40	5.4% *N* = 51
	Extremely severe (20–42) *N* = 127	6.4% *N* = 50	8.1% *N* = 77
DASS stress	Normal (0–10) *N* = 1,199	71.4% *N* = 558	67.8% *N* = 641
	Mild (11–18) *N* = 126	6.1% *N* = 48	8.2% *N* = 78
	Moderate (19–26) *N* = 171	8.7% *N* = 68	10.9% *N* = 103
	Severe (27–34) *N* = 176	10.0% *N* = 78	10.4% *N* = 98
	Extremely severe (35–42) *N* = 56	3.8% *N* = 30	2.7% *N* = 26

* *Defining ranges of scores for each category given in brackets*.

### Regression Models to Compare Depression, Anxiety, and Stress (Between the Two Measurement Points of the Second Wave)

#### Depression

The results of the regression stepwise model for depression are summarized in Table 4. The highly significant *p*-value for the time point in the zero-inflated part of the model indicates that an increased proportion of people not usually at risk for depression had an elevated risk for depression at the second time point (compared to the baseline—first time point). On the other hand, there was no difference between the two time points for those usually at risk for depression. Age (younger had higher odds of depression), satisfaction with finances, relationships, and housing significantly affected the odds of developing depression. The probability of being at risk for developing depressive symptoms and their magnitude were negatively associated with age, satisfaction with finances, relationships, and housing.

**Table 4:** Results of the ZINB regression analysis with DASS depression score as the dependent variable^*^.

	**Count model part**			**Zero-inflation model part**		
	***P*-value**	**Odds ratio**	**95% confidence interval**	***P*-value**	**Odds ratio**	**95% confidence interval**
Time point (Time point 1)	0.557	1.03	0.94–1.12	<0.001	0.62	0.46–0.82
Age	<0.001	0.99	0.99–0.99	<0.001	1.03	1.02–1.04
Satisfaction with finances	<0.001	0.94	0.91–0.97	<0.001	1.23	1.01–1.38
Satisfaction with relationships	<0.001	0.82	0.78–0.85	<0.001	1.73	1.45–2.07
Satisfaction with housing	0.002	0.93	0.89–0.97	0.015	1.24	1.04–1.47

* *For each categorical predictor, the level coded as the base for comparison is given in brackets*.

#### Anxiety

The results of the regression stepwise model for anxiety are summarized in Table 5. The significant *p*-value for the time point in the zero-inflated part of the model indicates an increased proportion of the people being at risk for anxiety compared to baseline—the first time point, while the significant *p*-value for the time point in the count part of the model suggests that the magnitude of anxiety symptoms among those at risk for anxiety was higher at the second measurement point of the second wave. Age (younger had higher odds for anxiety), and satisfaction with finances and relationships were associated with the probability of having anxiety symptoms. For those at risk for developing anxiety symptoms, the magnitude of symptoms decreased with age, satisfaction with finances, relationships, and housing, and was lower for men (compared to women).

**Table 5:** Results of the ZINB regression analysis with DASS anxiety score as the dependent variable^*^.

	**Count model part**			**Zero-inflation model part**		
	***P*-value**	**Odds ratio**	**95% confidence interval**	***P*-value**	**Odds ratio**	**95% confidence interval**
Time point (Time point 1)	0.027	1.13	1.01–1.25	0.019	0.75	0.59–0.95
Age	<0.001	0.99	0.98–0.99	<0.001	1.02	1.01–1.03
Gender (Male)	<0.001	1.27	1.11–1.46	0.273	0.84	0.63–1.14
Satisfaction with finances	<0.001	0.92	0.89–0.96	0.034	1.11	1.01–1.22
Satisfaction with relationship	<0.001	0.90	0.86–0.96	<0.001	1.57	1.35–1.83
Satisfaction with housing	<0.001	0.91	0.87–0.96	0.844	1.01	0.89–1.15

* *For each categorical predictor, the level coded as the base for comparison is given in brackets*.

#### Stress

The results of the regression stepwise model for stress are in Table 6. The significant *p*-value for the time point in the zero-inflated part of the model indicates an increased proportion of the population having an elevated risk for developing symptoms of stress compared to baseline. On the other hand, the *p*-value for the time point in the count model part indicates no difference in the level of stress symptoms between the time points for those usually at risk for stress. Age (younger had higher odds of stress), and satisfaction with relationships and housing were associated with risk for developing stress symptoms. The odds of being at risk for developing stress symptoms were also higher among health workers (compared to people working outside of the health sector). For those at risk for developing stress symptoms, the magnitude of symptoms decreased with age, satisfaction with finances, relationships, and housing, and was lower for men (compared to women).

**Table 6:** Results of the ZINB regression analysis with DASS stress score as the dependent variable^*^.

	**Count model part**			**Zero-inflation model part**		
	***P*-value**	**Odds ratio**	**95% confidence interval (lower bound–upper bound)**	***P*-value**	**Odds ratio**	**95% confidence interval (lower bound–upper bound)**
Time point (Time point 1)	0.539	1.02	0.95–1.11	0.005	0.66	0.49–0.88
Age	<0.001	0.99	0.99–0.99	<0.001	1.02	1.01–1.03
Gender (Male)	0.004	1.15	1.05–1.27	0.163	0.79	0.56–1.10
Health worker (Yes)	0.457	1.03	0.95–1.12	<0.001	1.69	1.24–2.31
Satisfaction with finances	0.011	0.96	0.93–0.99	0.473	1.04	0.93–1.17
Satisfaction with relationship	<0.001	0.87	0.83–0.90	<0.001	1.83	1.51–2.21
Satisfaction with housing	0.006	0.95	0.92–0.99	<0.001	1.35	1.13–1.61

* *For each categorical predictor, the level coded as the base for comparison is given in brackets*.

## Discussion

Based on the data from Slovenia, the lasting effect of the COVID-19 pandemic and social distancing is likely to present a profound threat to the psychological health of the general population. The findings of this study show that the second wave was associated with higher risk for depression, anxiety, and stress. The risk for all three was higher for younger participants; among the population at risk, the younger participants also showed higher levels of DAS. Women showed a higher risk for anxiety and stress, but regarding depression, no gender differences were found. Finances, relationships, and housing dissatisfaction were relevant predictors of DAS symptoms and were also associated with higher levels of DAS. Health workers in our sample showed a higher risk for stress, but not for depression or anxiety, than the general population.

The first wave of COVID-19 pandemic presented a distressing situation, full of uncertainty and the need for adaptation. After the first wave, life returned to more normal levels and the pandemic situation looked manageable and transient. In the fall months, however, the second wave of COVID-19 arose throughout Europe, and the number of infections was higher each day. The world seemed no longer predictable and safe; unemployment rates were rising, changes in different restrictive and adaptation measures were fast, and the number of new COVID-19 infections suddenly grew exponentially, in contrast to the situation at the beginning of the pandemic. We can hypothesize that, for our sample, all of these factors contributed to feelings of anxiety, stress, and depression that could pose a higher threat for mental health in predisposed people. Similarly, the Italian study that analyzed the mental health impact following weeks of exposure to the pandemic, and the related containment measures, found that the symptoms of depression, anxiety, and stress tended to increase over time (3).

According to meta-analyses, the prevalence of stress, anxiety, and depression in the general population as a result of the COVID-19 pandemic is around 30% (27–29). In our sample, similar or higher rates of depression, anxiety, and stress in were observed in at both time points. 35.2% of respondents reported at least mild depressive symptoms even at baseline of second wave of the pandemic; 20% of people in our sample reported moderate to severe and >5% extremely severe depressive symptoms. Furthermore, 21–26% of participants in our sample reported moderate to severe/extremely severe levels of anxiety, and 22–24% moderate to severe/extremely severe symptoms of stress. At the second time point, 31% of people in our sample showed at least mild levels of anxiety on DASS-21, 26% of those moderate to severe/extremely severe anxiety symptoms, which is comparable to the 23.4% mean anxiety prevalence reported in COVID-19 studies measured with the DASS-21 scale (30). The results need to be interpreted in light of the national data, especially given the low prevalence of depression previously found in studies on the Slovenian population. “Predict D” study found the incidence of depression followed over 24 months was lowest in Slovenia (5.3%) among the participating European countries (UK, Spain, and Portugal); the prevalence of depression and anxiety was expectedly higher for women compared to men (31). Two other observational studies on a representative sample of family medicine practice attendees in Slovenia found a similarly low prevalence of major depression, 5.8 and 3.4%, respectively, and also a low prevalence of anxiety syndromes of 2.7% (32, 33). These data are nevertheless somewhat surprising, especially considering the high suicidality rates in Slovenia.

Several studies have shown that age may be an important factor in predicting mental health, especially depression, during this pandemic (34). Moderate to extremely severe levels of anxiety, depression, and stress were found on DASS-21 by 21–34% of the university students during the first weeks of confinement (35). Among the proposed stressors for poor mental health in younger adults, study obligations, finances (rents, career prospects, job instability), and lower living security, as well as social distancing and limited social interactions were exposed, which may have further exacerbated stress (30, 36).

Consistent with previous studies, the women of our sample coped with the pandemic situation, as regards mental health, in a considerably worse way than men (7, 37, 38). However, in contrast with a few other studies (39), we found no gender differences regarding the risk for depression, even though levels of anxiety and stress were higher among women in our sample. In comparison, a recent systematic review with meta-analysis (40) showed no gender differences regarding depression or anxiety during this pandemic and the gender differences data among studies is still inconclusive. Given these surprising results, studies conducted during the COVID-19 pandemic have found that males and females experience stressors in similar ways (41), and we can speculate that the COVID-19 pandemic presents such an important stressor that might mitigate—at least for a part of the population—other risk factors for depression and contributes to this gender gap.

Women are also generally more likely to be affected by the social and economic consequences of the pandemic, mostly due to less secure employment than their male counterparts. However, in Slovenia, for the last few years, despite a prolonged crisis, the gender labor income gap has been among the lowest in the Organization for Economic Co-operation and Development (OECD), reflecting the relatively low wage gender gap and a high employment rate for women, where Slovenia is at the top of the EU-28 among all member states. In 2019, 88% of women in Slovenia with two children were employed, followed by Sweden in second place with 87% (42). We can speculate, that at least from that perspective, those changes were less marked for the women in our sample and presented less risk for depression.

In the present study, satisfaction with finances, relationships, and housing proved relevant for DAS, as predicted. Considering the known association of personal conflicts and dissatisfaction with close relationships as a relevant predisposing factor in mood and anxiety symptoms (43), participants in unstable relationships showed higher levels of depression and anxiety than those in stable relationships (44), and satisfaction in partnership reduced the risk of depression and anxiety (45, 46).

Regarding health workers, our study showed a significantly higher risk for stress but no difference regarding the risk for depression or anxiety compared to non-health workers of our sample, which is surprising, given the extraordinary pressure, increased workload, physical exhaustion, transmission risk, and the need to make ethically difficult decisions on the rationing of care; all of which may have dramatic effects on their physical and mental well-being and also, given the high prevalence of DAS reported among health care workers during the beginning of this pandemic (38, 47). However, relatively few studies compared the DAS in health workers to the general population. A recent systematic review compared mental health problems between health care workers and other populations affected by COVID-19 and showed no significant differences in depression or anxiety (40). Furthermore, another systematic review with meta-analysis showed that the proportion of depression in nurses and medical doctors during the COVID-19 pandemic was similar to that found in the general population (48). These results are also consistent with previous studies that have shown that during epidemics and crises (e.g., SARS, Ebola), health care workers generally have the same level or fewer mental health problems than the general population (47, 49). One way of explaining the difference could be better knowledge of the COVID-19 disease as well as economic and job security, in contrast to several other professions during the second wave, when people were bound to their home, and many lost their source of income due to closing of all public and commercial activities and sectors except industry. Health workers were not experiencing the home confinement, and we can speculate that the associated risk factors (relationships, financial state, and housing) did not present an additional threat to mental health. Nevertheless, depressive symptoms commonly appear when the acute crisis is over. As health workers in Slovenia were fully recruited to work on COVID and non-COVID wards and outpatient services and showed a higher risk for stress in our study data, longitudinal studies will show whether depression in this population would rise when the pandemic-related health crisis is over.

The rise of the second wave of COVID-19 with corresponding measures that limited people to home confinement was associated with higher odds of anxiety and depression. We can speculate that home confinement and limited interactions with others (to the basic, close relationships) elevated the risk for depression and anxiety in vulnerable people. The second wave of the pandemic in Slovenia, with its exponential rising rates of infections, could have presented a threat to mental health and a higher risk for depression, anxiety, and stress. However, we lack the data from the first spread of infections and longitudinal studies will have to confirm, whether the mental health of the population deteriorates, as the pandemic continues.

Our study has several strengths. To the best of our knowledge, this is the first study carried out during the second wave of the COVID-19 pandemic in Slovenia with a large sample from the general population. So far, one study investigated psychological functioning, mental health, and stress among Slovene adults at the beginning of the COVID-19 outbreak (during the first five days of the declared epidemic in Slovenia) and found that women, younger, and less educated participants had higher odds for less favorable psychological functioning during the COVID-19 outbreak (50).

Even though the design of our study is not longitudinal, it allows for comparison between the timeframes, given the limitation that the research has been done on two independent samples, that are not adjusted. Furthermore, validated and reliable assessment instruments have been used, and the use of DASS-21 to evaluate depression allows direct comparisons between countries (3) and adds to the body of literature. The benefit of our study is also a large sample from the Slovenian population. Some limitations of this study need to be noted. We are aware that using an online tool is not the best methodological choice since it may have excluded some of the elderly population and the snowball sampling carries a risk for selection bias. However, with person-to-person contacts restrictions in place, we nevertheless reached a large sample in a relatively short time. The self-selected sample may also compromise the generalisability of our results as the people more vulnerable to anxiety may have been more prone to participate. Furthermore, the design of this research does not allow conclusions about causality. Even though our study searched for depressive or anxiety symptoms, which we cannot interpret as a diagnosis/disorder, attention and screening of the population should be considered.

## Conclusions

The COVID-19 pandemic has lasted and reached worldwide dimensions. Based on the data from Slovenia, the second wave of the COVID-19 pandemics was associated with a higher risk for depression, anxiety, and stress, especially for the younger population. Analyzing mental health during the waves of the pandemic provides an important contribution to the field in order to further clarify the pandemic-related changes on mental health. With a concomitant rise of the new variants of SARS-CoV2, times remain unpredictable. Even with the vaccination progress, the pandemic is ongoing, and the pandemic-related mental health burden can be expected to be growing.

Our findings highlight the urgent need for offering mental health services and tailored interventions to reduce the mental health burden. Attention should be given to younger age groups and their satisfaction with basic living conditions (housing, relationships, and financial position) when assessing mental health in primary and clinical settings. Future work is necessary to understand the longitudinal effects on mental health and develop effective interventions as the pandemic continues.

## Data Availability Statement

The datasets presented in this article are not readily available because the data is not publicly available as it was collected with the understanding that only aggregated and anonymized data will be published. Requests to access the datasets should be directed to Polona Rus Prelog, polona.rus@psih-klinika.si.

## Ethics Statement

The studies involving human participants were reviewed and approved by the Republic of Slovenia National Medical Ethics Committee under protocol No. 0120-283/2020/7. The patients/participants provided their written informed consent to participate in this study.

## Author Contributions

PR designed the article, with input from PP and AS. PR carried out the literature search and drafted the manuscript. PP contributed to interpretation of the data. PP and AS supervised the writing of the article. TM performed the main statistical analysis and contributed to the design, writing, and proof reading of the paper. AS contributed to the statistical analysis, data acquisition, design, writing, and proof reading of the paper. All authors contributed to the article and approved the submitted version.

## Funding

TM and AS have received support from the Slovenian Research Agency (ARRS) as members of the research program Artificial Intelligence and Intelligent Systems (Grant No. P2-0209).

## Conflict of Interest

The authors declare that the research was conducted in the absence of any commercial or financial relationships that could be construed as a potential conflict of interest.

## Publisher's Note

All claims expressed in this article are solely those of the authors and do not necessarily represent those of their affiliated organizations, or those of the publisher, the editors and the reviewers. Any product that may be evaluated in this article, or claim that may be made by its manufacturer, is not guaranteed or endorsed by the publisher.
