# Supplementary material for: Risk of Depression, Anxiety, and Stress During the Second Wave of COVID-19 in Slovenia
Source: Front Psychiatry. 2022 Jan 12;12:788898. doi: 10.3389/fpsyt.2021.788898 (PMC8791306; doi:10.3389/fpsyt.2021.788898)

Research problem and design

- The coronavirus disease 2019 (COVID-19) pandemic has led to numerous negative consequences on mental health of the population throughout the world.
- **Main aim:** comparing the risk for depression, anxiety, and stress during two points of the second wave of the epidemic in Slovenia (baseline time point and height of the second wave).

- **Variables**:
  - Dependent: Depression, Anxiety, Stress (DASS-21)
  - Independent: Gender, Age, Healthcare Worker (Y/N), Satisfaction with Finances, Housing and Relationships

Sample and Analysis

- **N=1790** (Time point 1=782, Time point 2=946, 62 excluded from the analysis as the "buffer zone")
- Online survey, snowball sampling of general population
- Zero-inflated binomial regression analysis and Mann-Whitney U-test

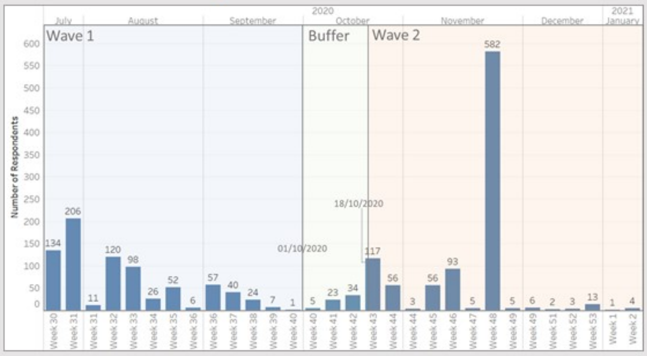

Findings

- Height of the second wave was associated with higher levels of depression, anxiety and stress
- The risk of all three was higher for younger participants
- Women showed a higher risk for anxiety and stress
- Health workers in our sample showed a higher risk for stress than the general population

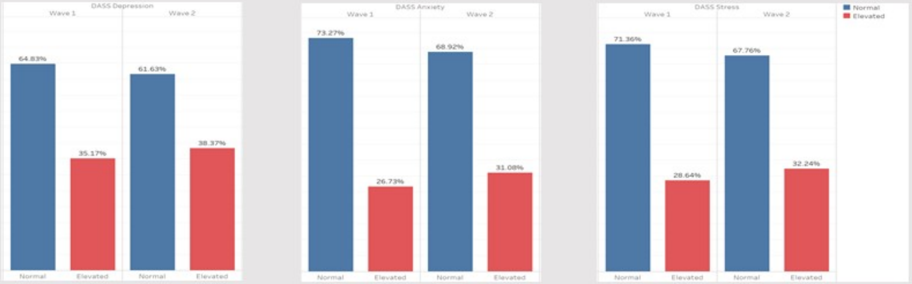

Supplement: Supplementary file 1 [file Data_Sheet_1.PDF]
